# Unexpected profiles in vasectomy demand during a national campaign in Bolivia: cross-sectional study and associations in age, number of children, and motivations for seeking vasectomy

**DOI:** 10.1186/s40834-026-00441-3

**Published:** 2026-03-25

**Authors:** Alison T. Hoover, Samuel Lawton, Silvia Velasco Parihuana, Patricia Lledo Weber, Michel Labrecque, Dominick Shattuck, Ana Cecilia Velasquez Rossi

**Affiliations:** 1 World Vasectomy Day, New York City, NY USA; 2 MSI Reproductive Choices Bolivia, La Paz, Bolivia; 3 MSI Reproductive Choices, London, UK; 4 Department of Family and Emergency Medicine, Laval University, Quebec City, QC Canada; 5 Johns Hopkins University School of Medicine & Bloomberg School of Public Health, Baltimore, MD USA

**Keywords:** Vasectomy utilization, Male contraception, Family planning services, Cost barriers, Gender norms, Bolivia, Masculinity

## Abstract

**Background:**

Vasectomy utilization has historically been very low in Bolivia, constituting just 0.1% of the contraceptive method-mix. To address one of the perceived barriers to vasectomy utilization—cost—MSI Reproductive Choices Bolivia partnered with World Vasectomy Day, No-Scalpel Vasectomy International, and Laval University in 2021 to train in-house providers, and reduced the previous cost of contracting private urologists by half. Demand for procedures during the training campaign was considerably higher than expected; although just 77 vasectomies were provided in 2019, 882 men contacted MSI Bolivia in 2021 to request appointments in the one-month campaign.

**Methods:**

A cross-sectional survey of vasectomy clients was implemented post-procedure to capture demographic characteristics as well as to assess motivations for getting a vasectomy.

**Results:**

Among the 262 vasectomy clients who completed the survey, the average age was 31, and half (50.4%) of respondents had one or two children, 3.1% had four or more, and 30.5% had none. Motivations varied by client type with most clients reporting “wanted only the number of children they had”, while higher percentages of young clients who were often without children reported the “costs of children”, “overpopulation and the environment”, and “violence and social unrest” as their top motivations.

**Conclusions:**

The results of this study and the related vasectomy campaign reflected a high unmet demand for affordable vasectomy services in Bolivia. The relatively high proportion of single and/or childless men under 30 years old in the sample may also reflect a larger societal shift in pregnancy intention among younger generations and merit further investigation. These results have implications for demand-generation strategies and pre-vasectomy counseling, and may reflect a market for reversible male contraceptive technologies still in development.

## Background & Introduction

Vasectomy, or male sterilization, is a safe, effective, relatively inexpensive, and permanent form of contraception with few complications [1]. It is also one of only two modern contraceptive methods available to men to control their fertility. However, globally less than 2% of women using contraception rely on vasectomies [2]. Typically, vasectomy uptake is more prominent in high-income countries, with a 3% prevalence compared to 0.6% prevalence in low-income countries [3]. In Bolivia, vasectomies are just 0.1% of the contraceptive prevalence, comparable with neighboring Peru (0.4%), Chile (0.1%), Argentina (0.2%), and Paraguay (0.1%) [4].

MSI Reproductive Choices (formerly known as Marie Stopes International) is a global organization that provides contraceptive services and products, including vasectomy, in 37 countries around the world. In Bolivia, MSI operates eleven centers and six mobile units offering a comprehensive suite of contraceptive and other sexual and reproductive health services.

Before 2021, MSI Bolivia contracted vasectomy services to external private practice urologists, which increased the price for clients and resulted in a vasectomy costing twice as much as tubal ligation at 1,500 BOL, or 217 USD. This is an important distinction as it represents an inverse of the global norm; as a significantly less invasive procedure, the costs of a vasectomy are traditionally half the costs of a tubal ligation [5]. In hopes of reducing the cost and making vasectomy more accessible, MSI Bolivia, two US-based non-governmental organizations World Vasectomy Day and No-Scalpel Vasectomy International, and Laval University in Quebec, Canada, organized a provider training for MSI Bolivia general practitioners in 2021 to make the procedure available through MSI providers and eliminate the high fees charged by external urologists.

Before 2020, demand for vasectomy as a contraceptive method had been on a global decline [3, 6]. Declines in vasectomy demand have been attributed to low awareness among potential users, poor health-seeking behavior among men, limited focus and prioritization among governments and service providers, and insufficient supply of trained providers [3, 7, 8]. The COVID-19 pandemic, declared by the World Health Organization from January 2020 to May 2023, and interrelated social, economic, and political unrest reshaped health-seeking behaviors including increased contraceptive demand and a drop in desired family size [9–12]. This shift in fertility desires during economic crises or epidemics is well-documented and typically articulated by women, who manage the burden of contraceptive responsibility worldwide [10, 13, 14].

Ethnographic evidence from Santa Cruz, Bolivia, suggests that narratives blaming men’s health behaviors on “machismo” may influence men’s engagement with vasectomy services. Within HIV care, machismo has been shown to operate as a moralizing frame that portrays men as irresponsible, contributing to delayed care seeking and disengagement. Applied to vasectomy, this framing can obscure men’s diverse understandings of masculinity and reproductive decision-making, while increasing provider doubt toward men seeking permanent contraception. These dynamics point to the need to reframe vasectomy services in ways that reduce stigma, recognize male reproductive agency, and support equitable access [15].

Demographic trends for men’s contraceptive use are not well documented outside the US, but US findings have identified a recent shift. One study, marking a considerable increase in vasectomy demand in the US after the Supreme Court reversed the federal right to abortion, demonstrated changes in the demographic characteristics of men seeking vasectomy. The median age of men seeking vasectomy after the Dobbs ruling was significantly younger, resulting in a distinct increase in clients who were under 30 and childless [16–18]. In Bolivia, abortion is illegal with some exceptions in cases of rape, incest, or if the pregnancy poses a severe risk to the health or life of the pregnant person [19].

There is a need to examine demographic trends in vasectomy demand and document the impact of price reductions on vasectomy uptake. This study examines the demographics of a sub-sample of individuals receiving vasectomies during a campaign conducted by MSI Bolivia, and whether age and parenthood of vasectomy users were associated with their motivations and fears towards this family planning method.

## Methods

### Vasectomy provision: provider selection, training, and clients

A rigorous competency-based selection process was used by MSI Bolivia to identify four of their providers from different regions in the country, who were trained by two master vasectomy trainers in La Paz, Bolivia in November 2021 [20]. Training included didactic presentations and overviews, practice on realistic silicone vasectomy simulators, and supervised implementation of the procedure in accordance with training standards [21]. Each provider completed a minimum of 30 supervised vasectomies before attempting independently.

**Fig. 1:**
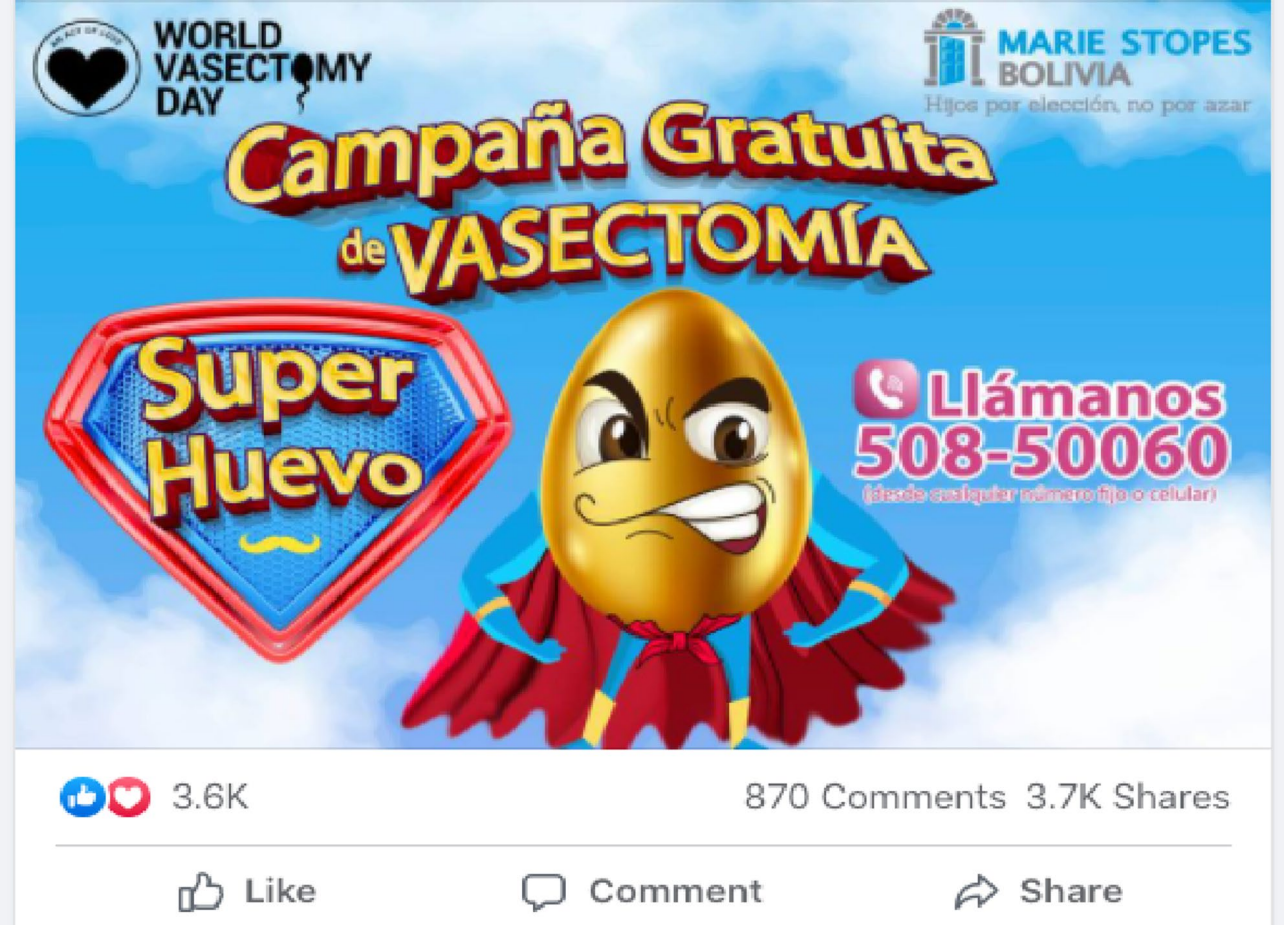
Example of Facebook advertisements for the initial 2021 vasectomy campaign

Vasectomy client recruitment took place through a social media campaign, which announced the free service (Fig. 1). Social media use is very high in Bolivia, with 95.3% of the population over 13 years of age using a social media platform, making it an effective platform for reaching men across demographics [22]. MSI Bolivia already had a strong existing social media following, with over 200,000 followers on both Facebook and TikTok. MSI Bolivia first started advertising their vasectomy services on social media in 2020, when they had their first campaign while contracting out to external urologists and with reduced services during COVID-19 pandemic-related closures.

The 2021 social media campaign included both organic and paid promotion, as is standard for MSI Bolivia’s service promotion. An initial amount was invested in one week of social media advertisements (116 USD). Initially, the audience was not segmented, and advertising targeted men and women. The existing MSI Bolivia following was 90% women, who generated immense engagement through engaging with the campaign. The second phase of the one-week advertisement targeted men between 20 and 45 years old who had searched for information on gyms, food, universities, music, and nightlife in the cities where MSI Bolivia had services available. No additional funds were used to target advertisements after the initial one-week investment.

The social media campaign resulted in high volumes of calls requesting appointments. The increased interest in vasectomies can be seen by comparing the 77 vasectomies provided by MSI Bolivia’s contracted urologists throughout the entire year of 2019, against the engagement during this one-month campaign when 882 men contacted MSI Bolivia for appointments, 704 initial consultations were provided, and 503 vasectomies were performed (Table 1). Figures from 2020 are not generally used for comparison as clinic closures related to the COVID-19 pandemic skewed the data.

Campaign appointments varied by site, but spanned approximately seven weeks starting October 8, and ending December 20, 2021, and included 70 days of vasectomy provision, averaging more than seven vasectomies per clinic day. This type of campaign effectively increased vasectomy demand, as MSI Bolivia conducted a similar campaign the following year and the number of vasectomies increased almost two-fold to 918 vasectomies. However, there is only relevant demographic survey data from the 2021 campaign.

**Table 1:** Number of vasectomy-related procedures scheduled and completed by site in 2021

Clinics	Cochabamba	La Paz	Chuquisaca (Sucre)	Santa Cruz	Total
Scheduled Counseling Sessions	237	452	20	173	882
Attended Counseling Sessions	202	347	19	136	704
Scheduled Procedures	172	265	15	111	563
Completed Procedures	151	246	11	95	503

### Study design and population

A cross-sectional study was designed to describe the demographic characteristics and motivations of men who received vasectomy services through the MSI Bolivia 2021 vasectomy campaign. The data collection tool was self-administered via mobile phone using questions adapted by the MSI Bolivia team from a previous survey implemented by Population Council Mexico in collaboration with World Vasectomy Day in 2017 [23]. This research was reviewed and approved by the independent Ethics Committee of MSI Reproductive Choices (approval number: 003–22). Of note, Bolivian IRB approval was not available, as ethics committees were not reviewing sociobehavioral research protocols during the pandemic. Some additional aggregate data from clinic medical records is presented as supplementary findings to assist in contextualizing the sample’s age demographics in ongoing demand demographic profiles.

### Data collection

The consent process for the procedure and the survey were separate. Procedural consent included a 15-minute counseling session up to one week before the procedure, which was reviewed again on the day of the procedure by the provider, who also solicited client signatures. Survey consent was administered by trained clinic receptionists on arrival at the clinic. In addition to consent, participants were informed that participation in the survey was not a requirement for receiving a vasectomy that day. After their procedure, participants who signed the consent form were provided a QR code that brought them to the Google Forms survey. They were encouraged to complete the survey on their phone in the recovery area. The survey had exclusively closed-ended questions that queried demographics and motivations for seeking a vasectomy. Ultimately, 436 vasectomy clients consented (87%) and 262 self-completed the survey (52% of possible sample) across the four campaign sites in Bolivia: La Paz, Cochabamba, Chuquisaca (Sucre), and Santa Cruz.

### Analysis

Data captured in the Google Form survey were reviewed by the study team and downloaded for translation, formatted in Microsoft Excel (2023), and cleaned and analyzed using R [24]. The R procedures included the tidyverse [25] and dplyr [26] packages in the analyses.

In the survey, respondents were asked to “select all that apply” to survey questions assessing motivations for vasectomy and fears regarding the procedure. They were not offered binary options of “yes” and “no”, and as such, the lack of indicated agreement cannot be used to suggest active disagreement. Respondents’ ages were dichotomized as either 30 and over or under 30 years old to allow for comparison with relevant literature [6, 16]. Respondents’ reported number of children was collapsed into four categories to consolidate observations and reduce the likelihood of reidentification. Given the small number of observations in Chuquisaca (*n* = 11), its data were combined with Cochabamba for analysis, which represents the three geological zones within Bolivia: highlands (La Paz), valley (Cochabamba & Chuquisaca), and plains (Santa Cruz).

Independence testing was performed through two series of Chi-square tests. The first series tested differences in responses to questions on vasectomy motivation, fears, and views on reproductive health by age (< 30/30+). The second series tested differences in respondents’ vasectomy motivation, fears, and views on reproductive health using children status (have children, yes/no). Statistical significance was assessed at α = 0.05.

## Results

### Demographics, vasectomy motivations, & fears

Table 2 presents the characteristics of the 262 vasectomy clients who completed the survey. Less than half of the respondents were under 30 years old (41.6%) and 9.9% were 40 years old and above. The average age of respondents was 31 ± 7.0 years old, ranging from 18 to 56. Most respondents (50.4%) had one or two children, while 30.5% had none, and 3.1% had four or more. Among the 109 men under 30 years of age, 60.6% did not have children. This proportion was 77.6% among the 65 men under 26 years of age.

Nearly three-quarters of respondents (79.4%) had completed some education beyond high school, and half (51.2%) had a college degree, which is higher than the Bolivian average of 30.8% [27]. Approximately half of all respondents were married (48.5%) at the time of completing the survey, while 10.7% were in a committed unmarried partnership, and 35.5% were single. One-third (34.7%) of respondents reported knowing someone else who had received a vasectomy, and most respondents received their vasectomy in either La Paz (45.8%) or Cochabamba/Sucre (40.1%) with a smaller proportion receiving their vasectomy in Santa Cruz (14.1%).

**Table 2:** Participant Characteristics (*n* = 262)

**Age (yrs)**	***n*** ** (%)**
18–25 26–29 30–34 35–39 40–44 45+	67 (25.6) 42 (16.0) 72 (27.5) 55 (21.0) 21 (8.0) 5 (1.9)
**Number of Children**	
None 1 2 3 or more	80 (30.5) 41 (15.6) 91 (34.7) 50 (19.0)
**Educational Attainment**	
At Least Some High School Associate’s Degree or Equivalent Bachelor’s Degree Specialist/MSc/PhD	54 (20.6) 74 (28.2) 107 (40.8) 27 (10.3)
**Marital Status**	
Single Cohabitating Married Divorced Other	93 (35.5) 28 (10.7) 127 (48.5) 13 (5.0) 1 (0.4)
**Known Someone with a Vasectomy**	
Yes No Did not answer	91 (34.7) 170 (64.9) 1 (0.4)
**City**	
La Paz Cochabamba & Chuquisaca (Sucre) Santa Cruz	120 (45.8) 105 (40.1) 37 (14.1)

Motivations and fears were compared across age groups (< 30 years, >=30 years) using a scale that allowed for multiple responses, including a “select all that apply”. Significantly more respondents under 30 years old reported their motivation as “overpopulation and the environment” and “violence and social unrest” than the older group (see Table 3). A greater percentage of older respondents reported limiting family size (“wanted only the number of children they had”) as their motivation. Comparisons of vasectomy fears did not reflect statistical differences across age groups, though a significantly higher percentage of younger respondents reported that they have a right to a vasectomy without consulting their partner (46.8% vs. 32.7%; *P*=.02) and a woman has a right to terminate a pregnancy if she chooses to do so (40.4% vs. 25.5%; *P*=.01).

Motivations and fears were also compared across child status (have children, yes/no) and revealed differences (see Table 3). A significantly higher percentage of non-parents reported the “costs of children”, “overpopulation and the environment”, and “violence and social unrest” as their top motivations. Predictably, significantly more respondents with children affirmed that their motivation was based on reaching their desired family size, and 17.6% of fathers reported they had more children than desired. Vasectomy-related fears revealed significantly more non-parents reporting they were not afraid to have a vasectomy (while a greater percentage of fathers reported fears related to pleasure and sexual performance). A higher percentage of childless men reported they have a right to a vasectomy without consulting their partner (56.3% vs. 30.8%; *P*<.001), their partner has the right to have tubal ligation without consulting them first (47.5% vs. 25.3%; *P*<.001), men and women are free to decide on their own which (non-permanent) method of contraception to use (75% vs. 58.2%; *P*=.0095), and a woman has the right to terminate a pregnancy if she chooses to do so (53.8% vs. 22%; *P*<.001). 

**Table 3:** Prevalence of common vasectomy motivations and fears among 2021 campaign vasectomy users by age and child status

Motivations	Age (years)				Child Status		
	< 30 *n* (%)	> = 30 *n* (%)	*P*-value*		No children *n* (%)	With children *n* (%)	*P*-value*
Additional child costs	37 (33.9)	38 (24.8)	0.11		35 (43.8)	40 (22.0)	< 0.001
Impact on overpopulation or the environment	36 (57.8)	31 (20.3)	< 0.001		61 (76.3)	33 (18.1)	< 0.001
Concerns about violence, social unrest	41 (37.6)	28 (18.3)	< 0.001		38 (47.5)	31 (17.0)	< 0.001
Because COVID affected my view of family planning	16 (14.7)	12 (7.8)	0.08		13 (16.3)	15 (8.2)	0.05
Already has more children than planned	12 (11.0)	21 (13.7)	0.51		1 (1.3)	32 (17.6)	< 0.001
You simply want only the number of children you have	35 (32.1)	101 (66.0)	< 0.001		10 (12.5)	126 (69.2)	< 0.001
**Fears**							
I was not afraid to have a vasectomy.	51 (46.8)	60 (39.2)	0.22		45 (56.3)	66 (36.3)	0.003
That the procedure would be very painful†	48 (44.0)	65 (42.5)	0.80		28 (35.0)	85 (46.7)	0.08
That I would become incontinent†	1 (0.9)	4 (2.6)	0.32		0 (0.0)	5 (2.8)	0.13
That I would enjoy sex less with my partner†	7 (6.4)	17 (11.1)	0.19		2 (2.5)	22 (12.1)	0.01
That my partner would enjoy sex with me less†	2 (1.8)	10 (6.5)	0.07		1 (1.3)	11 (6.0)	0.09
That my virility/masculinity/manhood would be diminished.†	8 (7.3)	19 (12.4)	0.18		6 (7.5)	21 (11.5)	0.32
That I would no longer be able to ejaculate or would ejaculate less†	11 (10.1)	21 (13.7)	0.38		3 (3.8)	29 (15.9)	0.006
That I would have to undergo general anesthesia†	6 (5.5)	13 (8.5)	0.36		4 (5.0)	15 (8.2)	0.35
Recovery time would be very painful†	0 (0.0)	1 (0.7)	0.40		0 (0.0)	1 (0.5)	0.51
Producing impotence†	10 (9.2)	25 (16.3)	0.09		7 (8.8)	28 (15.4)	0.15
That having sex was going to be painful†	7 (6.4)	12 (7.8)	0.66		4 (5.0)	15 (8.2)	0.35
That it would change my sexual preferences†	0 (0.0)	2 (1.3)	0.23		0 (0.0)	2 (1.1)	0.35

* Chi-square test

† False beliefs/common myths about vasectomy

### Previous vasectomy attempt

We compared the percentage of participants who reported seeking a vasectomy elsewhere, which was reported by one-third (32.8%). Younger men (under 30) were more likely to have sought a vasectomy before the MSI Bolivia campaign (38.5% vs. 28.8%; *P* = .097). Among this subgroup of men under 30, 61.9% cited cost as the reason they did not get their procedure. Other less common reasons for not getting a vasectomy when sought previously included time, insurance coverage, uncertainty, and vasectomy provider refusal.

### Ongoing vasectomy demand and age demographics

**Fig. 2:**
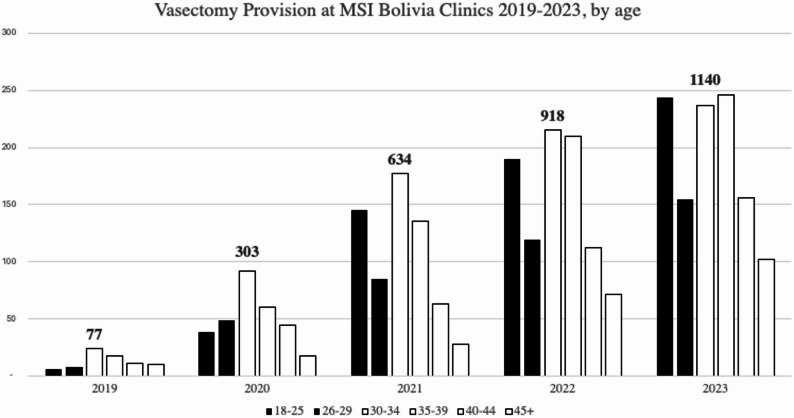
Vasectomy provision at MSI clinics from 2019–2023, by age of vasectomy client

As part of contextualizing the high demand and age demographics of the sample, aggregate records were pulled from MSI Bolivia clinic records (see Fig. 2). The records identify consistent growth in vasectomy demand and reveal similar age demographics to the study sample. Demand has steadily increased, nearly doubling annually from 2020 to 2022. There has also been consistent demand among men under 30 years of age, including outside of free campaigns, representing 36.3% of demand in 2021, 33.7% in 2022, and 35.0% in 2023. 

## Discussion

The results of this study and the related vasectomy campaign suggest a high unmet demand for affordable vasectomy services in Bolivia. The increase from 77 vasectomies in 2019 to 503 performed during the six-week campaign in November 2021 reflects this demand; the following year (2022), this number almost doubled at 918 vasectomy clients. Growth has continued annually since the 2021 provider training. Notably, while the number of both vasectomies and tubal ligations increased between 2019 and 2023, the ratio of vasectomies per tubal ligation changed dramatically, with more vasectomies than tubal ligations performed in 2023 [20]. The training activities implemented by MSI in combination with the campaign highlight the gap in the contraceptive method-mix for male-controlled methods in Bolivia. There is a need to better understand the increases in a younger vasectomy clientele and their motivations to meet Bolivia’s contraceptive demands and to understand if, how, and why some men’s desire for children or traditional families may be shifting.

This study highlights the considerable size of single and/or childless men under 30 years old (average age of the sample was 31 years old) who received a vasectomy through this campaign. These trends continued in subsequent service provision, with an average of 34.3% of vasectomy clients at MSI Bolivia in 2022 and 2023 under 30 years of age. These demographic phenomena of young and childless men were also noted in the US, Colombia, and Mexico in recent years, including data that pre-dated the COVID-19 pandemic [28]. This vasectomy trend may reflect a larger societal shift in pregnancy intention among younger generations [29]. In one US study, the average age of men seeking vasectomy is down to 35 from 38 [16], while in unpublished data from Profamilia in Colombia, 36.7% of vasectomy clients in 2022 were 30 years old or under (Diana Torres, personal communication, May 2023).

Childlessness was also a noticeable characteristic of the study respondents, with 30.5% of the sample reporting zero biological children. This characteristic was also present in a US study where they found the proportion of individuals seeking vasectomy without children jumped from 7.5% in 2002 to 12.4% between 2015 and 2017 [6], and then again to 16.9% in 2022 [16]. Similarly, the study of men seeking vasectomy in Mexico during a World Vasectomy Day event in 2017 found 17.2% of the sample reported no children [23]. Anecdotal conversations and notes from a research team member (AH) with seven childless participants provide additional context for the high rate of childlessness in the sample. The men described non-biological fatherhood (e.g. serving in a fathering role to their partner’s children from a previous relationship). They also cited social and economic contexts that made having a child feel irresponsible and could potentially lead to “suffering”, and noted that the pandemic’s volatility influenced them to remain childless. Environmental concerns were mentioned occasionally but were rarely the primary driver of the decision.

These anecdotal conversations, particularly around non-biological fatherhood, identified an underexplored segment of vasectomy decision-making requiring further examination. Current surveys focusing on biological children are not capturing the nuanced identity of non-biological fatherhood and its role in vasectomy decision-making. Further research should establish indicators to help identify this subset of vasectomy users, potentially through a more self-reported identity of “fathering” or “not fathering” as part of interpreting this seemingly growing trend of childless men seeking vasectomy.

Although the increase in vasectomy demand among young and childless men in Bolivia—and other countries—may reflect changing fertility desires among a subset of men, it may also reflect the campaign’s context and effectiveness in reaching a diverse audience and bringing in a new subgroup of vasectomy clients. Limited service availability during the pandemic may have also created a backlog of demand. Alongside these factors, eliminating client costs during the campaign likely made the procedure more affordable for younger individuals, who typically do not earn enough money to save for contraception. Before the training, a vasectomy at MSI Bolivia cost 1,500 BOL (217 USD), nearly an average month’s salary in Bolivia of 279 USD [30]. Since the training, the cost was halved to 850 BOL (123 USD) by eliminating fees charged by private urologists. This interpretation is supported by participants in this study who described cost as a barrier to completing the procedure when seeking services elsewhere.

It is important to note that MSI Bolivia has undertaken several approaches to facilitate broad and equitable access to vasectomy and other contraceptive methods; they continue to host annual vasectomy campaigns with free service provision nationwide, provide free services year-round in mobile units that operate in underserved peri-urban and rural areas, and operate a subsidy program for anyone who cannot afford the contraceptive method or services they are seeking, ensuring no one is turned away. In tandem with the overall price reduction, these approaches may contribute to drawing a younger demographic for vasectomy service provision.

This campaign trained several vasectomy providers within MSI Bolivia clinics, a well-recognized institution within Bolivia, using global MSI standards for training and client care. As mentioned above, men under 30 sought the procedure elsewhere and had been turned away before this campaign for several reasons, including staff unwillingness to perform the procedure. MSI Bolivia providers prioritize client autonomy, which may increase the likelihood that young and childless men may be over-represented among their clients [31].

The campaign utilized social media and other channels to advertise the services. This included directing paid advertisements on Facebook toward men between 20 and 45 years of age who had searched for information on gyms, food, universities, music, and nightlife. However, the organic reach of the advertisements (likes, shares, comments) of the campaign content caused the team to withdraw the funds, ultimately spending only 116 USD over a single week. Although social media is extremely common in Bolivia, the ads may have appealed to and reached younger demographics. Though global campaigns like those implemented by MSI and World Vasectomy Day provide robust examples of diversified marketing, this area of contraception would greatly benefit from systematic guidance on how to maximize the potential of social media to promote vasectomy uptake.

Anecdotally, men under 30 reported their motivations for vasectomy were influenced by the economy, violence and social unrest, and overpopulation and the environment. Characterizing the implications of the identified differences in the comparisons above is necessary to better understand the motivations for childlessness and potential demand for other forms of male contraceptives still in development. Future studies aligned with fertility preferences and contraceptive use among men should include demand-related questions about the delivery mechanisms, composition, and duration of future male contraceptives. Counseling guidelines inform clients that a vasectomy should be considered permanent, due to the limited number of skilled reversal providers, the high cost of reversal, and the low reversal success rate (approximately 50%).

Demand for vasectomy may shift with the continued availability of this method. However, the particularly high rates of childless men under 30 in the study sample and in the subsequent years of service provision at MSI Bolivia may also be indicative of shifts in norms related to virility and masculinity seen elsewhere [16, 28–29] and merit further investigation. Collectively, these data warrant further study about generational norm shifts related to fertility preferences, fatherhood, masculinity, and responsibility for contraception in Bolivia.

### Limitations

This study is not without limitations. As described above, slightly more than half (52%) of the vasectomy clients participated in the survey, and 60% of those who consented completed this survey. It is possible that the online format of the survey posed a barrier for some to complete the survey and may have biased the results. Feedback from all clients, as well as those who did not choose to receive a vasectomy, would have provided a more robust understanding of the context around this method and campaign. Not including an “other” option in the fears and motivations section may have missed additional fears and motivations among vasectomy clients. Other potential influences on demand for vasectomy and attitudes toward childlessness may include political unrest in Bolivia, economic downturns, and lockdowns related to the COVID-19 pandemic in October and November 2021.

## Conclusions

Whether or not the trends in this study shift or remain, the demand for vasectomy during the free campaign underscores a crucial need to make this service more affordable. Additionally, there is a need to closely examine demand demographics over time, including among young and/or childless men. These findings can inform guidance on adapting advertisements and pre-vasectomy counseling for clients across different age ranges. Relatedly, there is a need to reconsider the role of non-biological fatherhood in evaluating patient candidacy for vasectomy.

The pandemic and interrelated social and economic impacts have altered contraceptive access and fertility preferences. There is a need to consider the role that social upheaval plays in contraceptive autonomy and the implications for the ethics of sterilization access. Lastly, these men in the sample of young childless vasectomy clients may reflect a market for reversible male contraceptive technologies still in development.

## Data Availability

The datasets used and analyzed during the current study are available from the corresponding author on reasonable request.
